# Gold Coated Superparamagnetic Iron Oxide Nanoparticles as Effective Nanoparticles to Eradicate Breast Cancer Cells via Photothermal Therapy

**DOI:** 10.15171/apb.2018.024

**Published:** 2018-06-19

**Authors:** Ehsan Nassireslami, Morteza Ajdarzade

**Affiliations:** Department of Pharmacology &Toxicology, Faculty of Medicine, AJA University of Medical Sciences, Tehran, Iran.

**Keywords:** Fe_2_O_3_ nanoparticles, MUC-1 aptamer, MCF-7, NIR

## Abstract

***Purpose:*** Unique physiochemical properties of Fe_2_O_3_ nanoparticles make them great agents to serve as therapeutic and diagnostic nanoparticles (NPs). In this study, we developed gold coated Fe_2_O_3_ nanoparticles for photothermal therapy of breast cancer cells.

***Methods:*** Fe_2_O_3_ nanoparticles was prepared via microemulsion method and their surface was modified via gold. Differential light scattering (DLS) and transmission electron microscopy (TEM) methods were applied to evaluate physicochemical properties of NPs. Gold coated NP was further modified with MUC-1 aptamer as a targeting agent to increase drug delivery into the desired tissue. To evaluate cytotoxicity of prepared cells, MTT assay was employed. Targeting ability of aptamer modified NPs was assessed through confocal microscopy and flow cytometry method. Subsequently, MCF-7 and CHO cells were treated with aptamer modified NPs and were then irradiated via near infrared light (NIR) to produce heat.

***Results:*** The morphology of NPs was spherical and monodisperse with the size of 16 nm, which was confirmed via DLS and TEM. Confocal microscopy and flow cytometry results indicated that aptamer modified NPs had higher uptake compared to bare NPs. Finally, NIR exposure results revealed that higher uptake of NPs and application of NIR led to significant death of MCF-7 cells compared to CHO cells.

***Conclusion:*** To sum up, aptamer modified Fe_2_O_3_ nanoparticles showed higher uptake by cancerous cells and led to eradication of cancerous cells after exposure to NIR light.

## Introduction

Breast cancer is the most common type of cancer in women and the main cause of death for them.^1^ Early detection of cancer cells can significantly decrease mortality of cancer and will be beneficial for patients. SPIONs are important nano-carriers that have greatly attracted researchers’ attention due to their various functionality such as imaging, drug delivery, gene delivery, and hyperthermia.^2^ Because of their easy targeting characteristic by an external magnetic field, SPIONs can be used for the diagnosis and treatment of different diseases such as cancer and can be employed as drug-delivery carriers.^3,4^ The size and morphology of SPIONs play important roles in all their unique characteristics. The aggregation of SPIONs can occur due to their high surface-to-volume ratio and their magnetic property. These phenomena lead to their opsonizaion and elimination from the body. Therefore, it is fundamental to engineer the surface of SPIONs.^5^ The most common coatings layer for SPIONs includes gold and silica. These coating layers can stabilize SPIONs and decrease the erosion effect of the environment.^6^ Furthermore, surface modification of SPIONs with gold makes them suitable candidates for photothermal therapy of tumor due to their strong absorbance in the NIR electromagnetic spectrum.^7^ In addition to photothermal advantages, gold surface engineered SPIONs have low cell toxicity for normal cells.^8^ To improve the accumulation of NPs in the desired tissue, they can be conjugated with functional moiety such as antibody,^9-11^ peptide,^12^ aptamer ^13-18^ and folic acid ^19,20^ that can target overexpressed receptors onto cancerous cells. Among these targeting agents, aptamers have attracted great attention due to their favorable properties such as small size, low immunogenicity, low synthesis cost and high binding affinity.^21^ Due to these outstanding properties, aptamers have been employed as unique targeting moieties in nano-carrier systems. MUC-1 is a single stranded, DNA based aptamer that has been used as a targeting agent via different kinds of NPs on a broad range of epithelial cancer cells such as breast, colon, lung and prostate cancer.^22^

In this study, we developed SPIONs via microemulsion method and modified the surface of NPs with gold. The DNA based MUC-1 aptamer was conjugated on the surface of NPs to increase specificity of NPs delivery into cancerous cells. The targeting efficiency of MUC-1 conjugated NPs was assessed *in vitro* through confocal microscopy and flow cytometry. Finally, the therapeutic effect of gold coated NPs was investigated through irradiation of NIR light. To achieve this aim, cells were treated by gold coated NPs and their cell viability was examined after exposure to NIR light.

## Materials and Methods

Cethyltrimethylammonium bromide (CTAB), FeCl_2_.4H_2_O, FeCl_3_.6H_2_O, HAuCl, NH_2_OH.HCl, dithiotreitol (DTT), sodium citrate, MTT (3-(4,5-dimethylthiazol-2-yl)-2,5-diphenyltetrazolium bromide), Tris buffer acetate-EDTA (TAE), dimethyl sulfoxide (DMSO) and DAPI dye were purchased from Sigma, USA. Ethidium bromide and LysoTracker Red DND-99 were obtained from Life Technologies, USA. Human breast cancer (MCF-7) and Chinese hamster ovarian (CHO) cells were purchased from Pasteur Institute, Iran. DMEM medium and fetal bovine serum (FBS) were obtained from Gibco (Grand Island, USA). MUC-1 Aptamer as a targeting moiety was purchased from TAG Copenhagen A/S, Denmark. Amicon ultracentrifugal tube-Millipore (10 KDa) was purchased from Merck, Germany.

### Synthesis of SPIONs and its surface modification

Among different kinds of SPIONs, magnetite (Fe_3_O_4_) and maghemite (γ-Fe_2_O_3_) are two most common types of this class. To synthesize SPIONs in this study, microemulsion method was selected due to its simplicity and ability to produce monodisperse Fe_3_O_4_ nanoparticle. The synthesis of Fe_3_O_4_ nanoparticle was performed according to our previously reported procedure.^23^ Briefly, 202 mg of FeCl_3_ and 75 mg of FeCl_2_ with molar ratio of 2:1 were poured into 2 ml of deionized water (DI) to produce aqueous phase. Subsequently, the prepared aqueous phase was added to 25 ml of toluene and 1.8 g CTAB to produce the first microemulsion. The second microemulsion was composed of toluene (25 ml) and CTAB (1.8 g) plus 25% ammonium hydroxide solution (2.5 ml). After preparing the mentioned microemulsions, each one was separately mixed at 7000 rpm using a homogenizer and titrated via 1-butanol (approximately 1.3 ml) until the color of solutions became transparent. Subsequently, the prepared microemulsions were poured into a three-necked flask and homogenized at 7000 rpm under constant flow of N2 gas at 50 °C. After 60 minutes, the homogenizer was stopped and the product was cooled down to room temperature. 20 ml ethanol was added to the flask and the prepared Fe_3_O_4_ nanoparticles were separated via a magnet. Finally, separated Fe_3_O_4_ nanoparticles were washed 3 times with boiling ethanol, 2 times with acetone and DI water. After the washing steps, Fe_3_O_4_ nanoparticles were dispersed at DI water via ultrasonic probe.

The surface of Fe_2_O_3_ nanoparticle was modified via gold based on Xu et al.^24^ procedure with minor modifications. For this purpose, prepared Fe_3_O_4_ nanoparticles – which have little potency for gold coating – were first heated and exposed to air for 1 hour to be oxidized to γ-Fe_2_O_3._ At the first step of synthesis, HAuCl_4_·3H_2_O (2.5 mmol) was dissolved in chloroform (8 ml) and oleylamine (1 mmol). Subsequently, 8 ml chloroform solution containing 10 mg γ-Fe_2_O_3_ nanoparticles and oleylamine (1mmol) was prepared. At the next step, the HAuCl_4_ solution was poured dropwise into the γ-Fe_2_O_3_ nanoparticle solution. After 30 minutes, the gold coated γ-Fe_2_O_3_ nanoparticles were formed. By adding ethanol into the solution, the prepared gold coated γ-Fe_2_O_3_ nanoparticles were precipitated. To remove any residual, gold coated γ-Fe_2_O_3_ nanoparticles were washed 4 times with ethanol and hexane and dispersed in hexane. Finally, the gold coated γ-Fe_2_O_3_ nanoparticles were dried under vacuum and dispersed in aqueous solution which contains CTAB (0.1 M) and sodium citrate (0.1 mM) under sonication.

### Physicochemical characteristic evaluation of NPs

Size and zeta potential of γ-Fe_2_O_3_ nanoparticles were measured by DLS (Malvern Zetasizer Nano ZS, UK) at a scattering angel of 90 θ at 25 °C. For this purpose, γ-Fe_2_O_3_ nanoparticles were suspended at DI water (pH=7.4) and sonicated for 5 min before measurement. The morphology and exact size of NPs were examined via TEM method (Philips EM 208, 90kv). TEM samples were prepared by adding one drop of NPs solution on a copper grid with carbon film.

### Targeting of NPs with MUC-1 aptamer

MUC-1 aptamer with oligonucleotide sequence of 5'-HS-C_6_-GAG/ACA/AGA/ATA/AAC/GCT/CAA/GAA/GTG/AAA/ATG/ACA/GAA/CAC/AAC/ATT/CGA/CAG/GAG/GCT/CAC/AAC/AGGC-3' was applied. At first, thiol modified aptamers were activated by adding DTT and 10 KDa Amicon tube was used to separate the activated aptamers. Aptamers were then suspended in Tris Buffer (50 mM Tris, 100mM NaCl). Afterwards, the activated aptamer solution (50 µM) was added to 0.5 ml of NPs (50 µg/ml) and incubated while was shaking for 24 h. After 24 h, the conjugated aptamer was separated from the free aptamer through centrifugation at 18000 g for 20 min and was later suspended in Tris buffer.

### Agarose gel electrophoresis

Aptamer conjugation was examined by agarose gel electrophoresis (2% w/v). In order to run samples into agarose gel, free aptamer solution, aptamer-NPs solution and bare NPs solution were mixed with a loading buffer (SDS, 2-ME, bromo phenol blue, glycerol, Tris HCl) and poured into agarose wells and run for 20 min at 110 V. Afterwards, the gel was soaked in a 0.5% ethidium bromide solution due to its ability to attach oligonucleotide and was visualized by a UV Transilluminator (Peqlab biotechnology gmbh, Germany).

### Cell culture

MCF-7 and CHO Cells were cultured at 37 °C in 5% CO_2_ in DMEM medium supplemented with 10% FBS and 100 units/mL penicillin/streptomycin. The cells were seeded in 96 and 6 well plates and were grown to desired confluency.

### Cytotoxicity assay

MTT assay was used to examine cell toxicity of gold coated Fe_2_O_3_ nanoparticles. MCF-7 (MUC-1 positive cell line) and CHO (MUC-1 negative cell line) cells were seeded in 96 well plates (10000 cells per well) for 24 h in DMEM medium. Subsequently, the media were discarded and cells were treated with sterile NPs (conc. 10, 100 and 500 µg/ml) which had been suspended at free DMEM medium for 24 and 48 h. Later, the media were removed and each well was washed 3 times with PBS. Then, 100 µl of MTT solution (1 mg/ml) was added to each well and incubated for 2 h. Formazan crystals produced through addition of MTT reagent were dissolved by DMSO solvent and absorbance was read at 540 and 630 nm via ELISA reader (ELx800, BioTek instruments, USA). Six wells were used for each sample and cytotoxicity data were shown as mean ± SD. Mean absorbance of cells treated by NPs was divided by the mean absorbance of untreated Cell (control samples) to define cell viability of treated cells.

### Aptamer-NPs targeting efficiency evaluation via confocal microscopy and flow cytometry method

Targeting efficiency of NPs was evaluated via confocal microscopy. For this purpose, cover glasses were placed in 6 well plates and 0.1% gelatin solution was poured into each well. After 1 h, the remaining gelatin was discarded. Subsequently, MCF-7 and CHO cells were seeded in wells and after 48 h, the media were removed. Aptamer-NPs and bare NPs (100 µg/ml) were added to each well of plates and incubated for 8 h. Afterwards, the media were removed and cells were washed with PBS. NPs uptake was evaluated with LysoTracker Red, which is a specific dye for the acidic organelles. Higher entrance of NPs into cells is correlated with higher amounts of endosome and lysosome in treated cells and higher intensity of fluorescence.^25^ Treated cells were stained with LysoTracker Red dye (300 nM) for 1 h and afterwards the solution was discarded and the cells were fixed by adding 0.4% formaldehyde solution for 20 min. Finally, cells were stained with DAPI solution for 5 min to specify the nucleus and the cover glasses were transferred onto the glass slides and examined by Nikon confocal microscope A1 (Nikon Inc., Switzerland) armed with an A1 scan head and a standard detector using a 405 nm diode laser with DAPI filter and 543 nm diode laser (Melles Griot, USA) with TRITC filter. Furthermore, NPs uptake was checked by flow cytometry instrument to quantitatively calculate NPs entrance to cells. For this purpose, cells were cultured in 6 well plates and later incubated with aptamer-NPs or bare NPs for 8 h. After that, the NP suspension was removed and the cells were stained by LysoTracker Red for 1 h. After the staining step, cells were trypsinized, centrifuged and suspended in PBS and analyzed by flow cytometry (Partec PASII, Germany).

### Photothermal therapy 

MCF-7 and CHO cells were seeded in a 6 well plate (400000cell per each well) and were then incubated with 1 ml of aptamer-NPs (100, 200 and 500 µg/ml in DMEM medium) at 37 °C, 5% CO^2^ for 12 h. Afterwards, the NP suspension was removed, cells were washed 3 times with PBS and fresh DMEM medium was added. Finally, treated cells were exposed to NIR light (640-710 nm, 0.7 W/cm^2^) via LED for 1-5 min. After NIR light exposure, cell viability was examined by MTT assay.

## Results and Discussion

### Physicochemical characteristics of NPs

**Figure 1:**
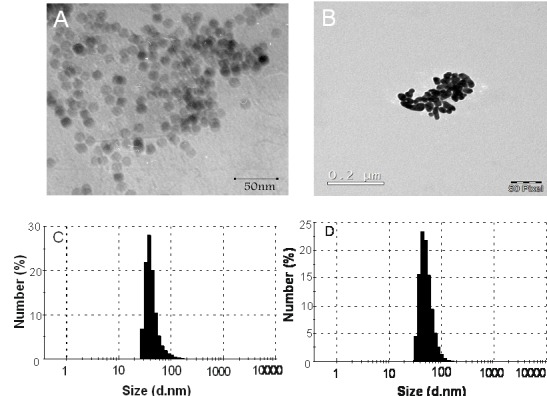
TEM images of (A) Fe_2_O_3_ nanoparticles and (B) Gold coated Fe_2_O_3_ nanoparticles. Size distribution analysis of (C) Fe_2_O_3_ nanoparticles, and (D) Gold coated Fe_2_O_3_ nanoparticles.

In recent years, promising physicochemical characteristics of SPIONs have made them outstanding nano-vehicles for different biomedical applications such as drug delivery,^26-28^ gene delivery,^29^ imaging,^30,31^ stem cell tracking ^32^ and hypothermia.^33-35^ Such favorable properties of SPIONs result from their small size. SPIONs with the size of less than 25 nm act as mono-domain magnets and show their magnetic properties when they are exposed to an external magnetic field. In order to produce NPs with favorable size, we employed the microemulsion method. DLS measurements and TEM results illustrated that the average size of the Fe_2_O_3_ nanoparticles was 16 nm, which increased to approximately 22 nm after formation of the gold layer (Figure 1). Zeta potential of Fe_2_O_3_ nanoparticles was -13mV and decreased to -17 mV after gold coating due to sodium citrate presence.

### Surface modification of NPs via gold and its evaluation by UV-visible spectroscopy 

**Figure 2:**
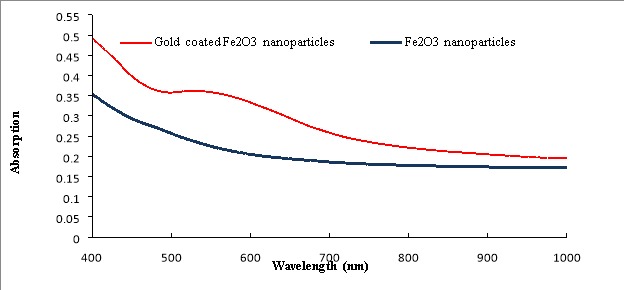
UV-visible spectra of Fe_2_O_3_ nanoparticles and Gold coated Fe_2_O_3_ nanoparticles. The peak around 580 nm belongs to the gold coating around nanoparticles.

The small size of SPIONs leads to large surface to volume ratio of these NPs and makes them vulnerable to aggregation.^36^ To overcome this drawback, gold layer ^37,38^ was employed. In order to conjugate targeting agents or drugs to SPIONs, we needed functional groups on the surface of SPIONs and gold coating provides this characteristic. The gold layer provides easy attachment with thiol conjugated agents; in addition, its steady shell around SPIONs protects them against oxidation events.^39^ Furthermore, the gold shell around NPs makes them sensitive to light absorption.^40^ The formation of gold layer around Fe_2_O_3_ nanoparticles was double checked via UV-visible spectroscopy. The noticeable peak around 520-590 nm was confirmatory evidence for gold layer formation. Figure 2 illustrates that for gold coated NPs, there is a peak at 580 nm area and bare NPs spectrum is free of this peak.

### Gel electrophoresis to examine conjugation of aptamer to NPs surface 

**Figure 3:**
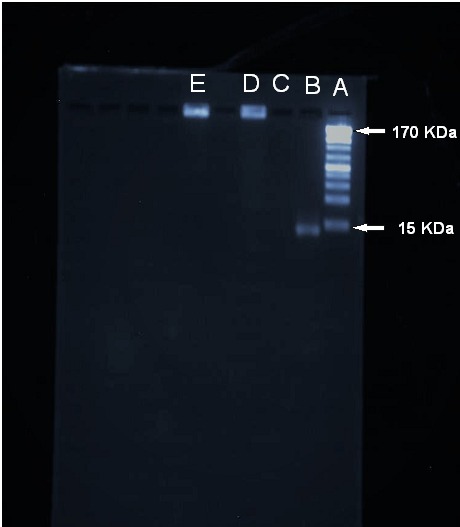
Evaluation of aptamer conjugation onto gold coated Fe_2_O_3_ nanoparticles via agarose gel electrophoresis. (A) Ladder, (B) Free MUC-1 aptamer, (C) bare NPs, (D) Aptamer-NPs and (E) Aptamer-NPs (high concentration)

Aptamer-NPs, bare NPs and free aptamer were run through agarose gel electrophoresis to examine aptamer conjugation to the NPs. Figure 3 depicts that after addition of mentioned samples to agarose gel wells, aptamer-NPs stayed at loading well and was stained by adding ethidium bromide. Furthermore, the free aptamer sample moved freely through agarose gel and was stained by ethidium bromide while no distinct band related to bare NPs was detected. Due to their larger size, Aptamer-NPs, bare NPs could not pass through gel pores and were trapped in the loading wells. These results confirmed aptamer conjugation onto the NPs.

### Cell viability of prepared NPs

**Figure 4:**
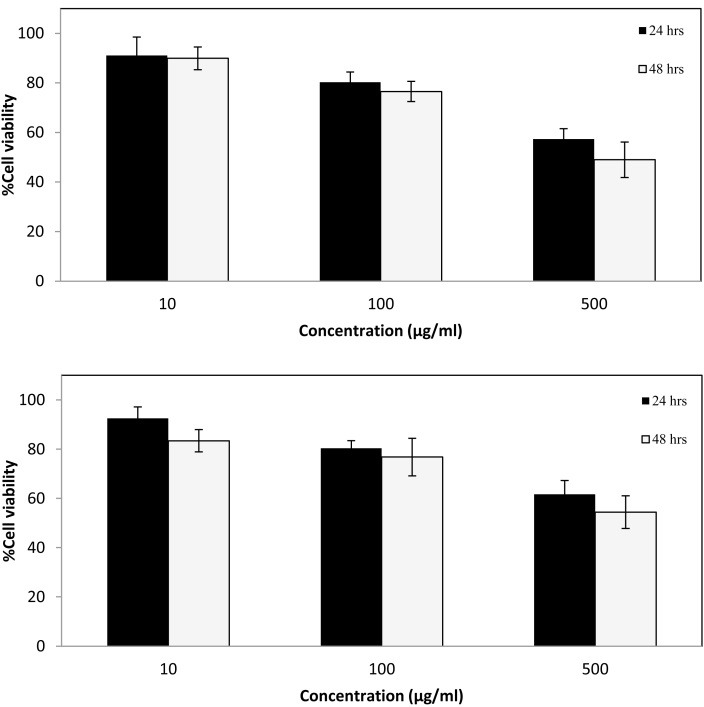
In vitro cell viability measured via MTT assay. (Top) MCF-7 and (Bottom) CHO cells were incubated with gold coated Fe_2_O_3_ nanoparticles (10,100, 500 µg/ml conc.) for 24 and 48 h (n=6, data is shown as mean ± SD)

Due to their biocompatibility, SPIONs have been widely used for drug delivery purposes. Surface modification of these NPs with gold increases their biocompatibility.^8^ Cell viability of prepared NPs was evaluated via MTT assay and results (Figure 4) indicated that 10 and 100 µg/ml concentrations of NPs have little effect on cell viability while increasing concentration to 500 µg/ml leads to more cell death.

### NPs uptake evaluation via confocal microscopy and flow cytometry

**Figure 5:**
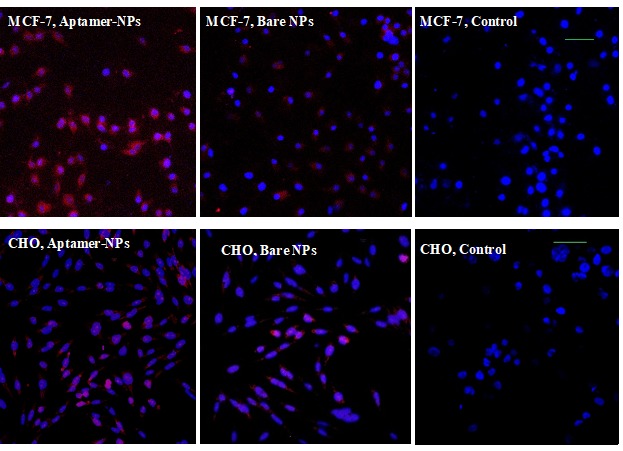
Confocal microscopy images of treated cells (MCF-7 and CHO cell line) with NPs. Lysosomes were marked by LysoTracker Red as an indicator of NP uptake, nuclei stained blue by DAPI. (Scale bars are 50 µm).

**Figure 6:**
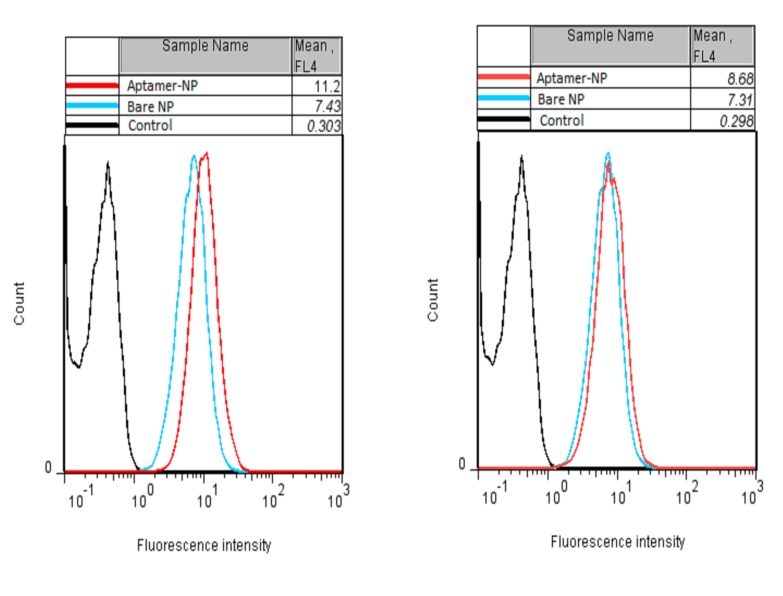
Flow cytometry results of cellular uptake of aptamer-NPs after staining of cells by LysoTracker Red as a marker of NPs uptake in (Left) MCF-7 (MUC-1 positive cell) and (Right)
CHO (MUC-1 negative cell)

The cellular uptake of aptamer-NPs and bare NPs was investigated by MCF-7 (MUC-1 positive cell line) and CHO (MUC-1-negative cell line). Overexpression of MUC-1 glycoprotein at the surface of epithelial cancer cells such as MCF-7 cells provides a great opportunity to apply MUC-1 aptamer on the surface of NPs as an efficient targeting moiety. Cells were treated by NPs and their cellular uptake was evaluated by LysoTracker Red as a marker of lysosome organelle. Cellular uptake of particles led to increase of lysosome and endosome, which makes these organelles indicator of cellular uptake.^25^ MCF-7 cells incubated with aptamer-NPs show higher fluorescent intensity compared to cells treated with bare NPs while aptamer-NPs and bare NPs show no difference in entering CHO cells (Figure 5). Furthermore, cellular uptake of NPs was quantitatively investigated by flow cytometry method (Figure 6). The flow cytometry results indicated that mean fluorescence intensity of aptamer-NPs and bare NPs were 11.2 and 7.4 in the MCF-7 cells, respectively. Flow cytometry results of CHO cells shows similar mean fluorescence intensity for aptamer-NPs and bare NPs (8.7 compared to 7.3, respectively).

This is strong confirmatory evidence of targeting efficiency of aptamer-modified nanoparticles. The conjugated MUC-1 aptamer efficiently mediate attachment of NPs to cancer cells and their internalization.^40^

### Cancerous cells eradication via photothermal therapy

**Figure 7:**
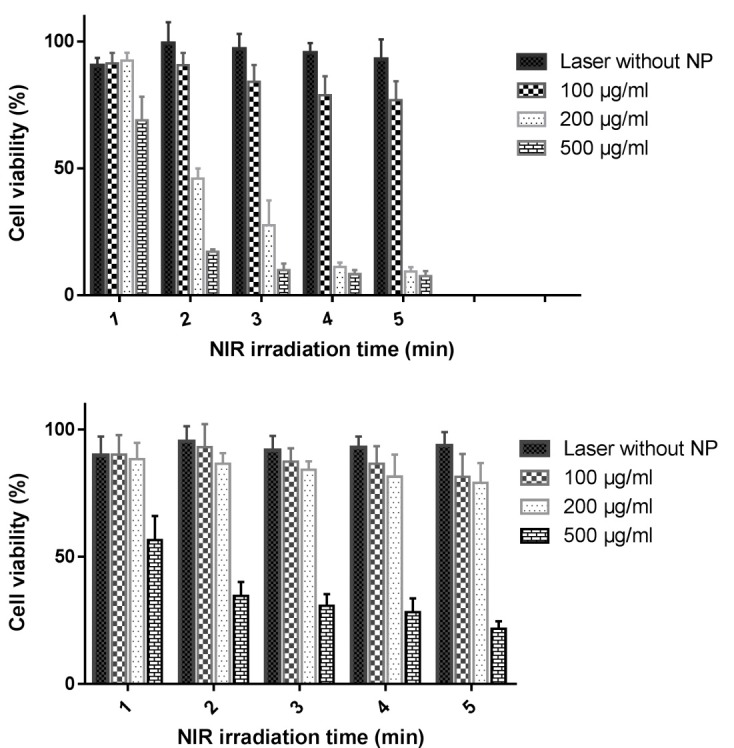
Cytotoxicity of (Top) MCF-7 and (Bottom) CHO cells treated with different concentration of aptamer-NPs after exposure to NIR light through 1 to 5 minutes (n=3, Mean ± SD)

During this step, MCF-7 cells were treated for 8 hours with aptamer-gold coated Fe_2_O_3_ nanoparticles (100, 200 and 500 µg/ml). They were then exposed to NIR light by using a light emitting diode (LED) for 1-5 minutes. Control cells were defined as cells that were exposed via NIR light without NPs treatment. After NIR irradiation, MCF-7 cells treated with aptamer-NPs experienced more cytotoxicity compared to bare NPs, a fact which resulted from photothermal effect of gold coated Fe_2_O_3_ nanoparticles (Figure 7). Internalization of gold coated NPs via desired cells makes them suitable targets for heat production after laser irradiation. While cancerous cells are more susceptible to heat, normal cells exhibit less susceptibility to heat, a fact which makes photothermal approach a safe and effective way to eradicate cancerous cells.^41,42^ NIR irradiation can lead to DNA damage,^43^ ROS mediated apoptosis,^44^ disruption of plasma membrane ^45^ or depolarization of mitochondrial membrane ^44^ and finally cell death.

## Conclusion

In summary, we have produced surface modified Fe_2_O_3_ nanoparticles and MUC-1 aptamer, as a targeted moiety conjugated onto gold layer around NPs. Confocal microscopy and flow cytometry method confirmed higher uptake of Aptamer-NPs compared to bare NPs in MUC-1 positive cells while aptamer-NPs and bare NPs exhibited similar cellular uptake in MUC-1 negative cells. In the final step, laser irradiation effectively eradicated cancer cells while normal cells experienced no harmful effects. Findings approve application of gold coated Fe_2_O_3_ nanoparticles as a biocompatible and efficient device in the field of cancer therapy.

## Ethical Issues

Not applicable.

## Conflict of Interest

The authors declare that they have no conflict of interest.

## References

[1] Torre LA, Bray F, Siegel RL, Ferlay J, Lortet-Tieulent J, Jemal A (2015). Global cancer statistics, 2012. CA Cancer J Clin.

[2] Xie J, Liu G, Eden HS, Ai H, Chen X (2011). Surface-engineered magnetic nanoparticle platforms for cancer imaging and therapy. Acc Chem Res.

[3] Hong SC, Lee JH, Lee J, Kim HY, Park JY, Cho J (2011). Subtle cytotoxicity and genotoxicity differences in superparamagnetic iron oxide nanoparticles coated with various functional groups. Int J Nanomedicine.

[4] Kocbek P, Kralj S, Kreft ME, Kristl J (2013). Targeting intracellular compartments by magnetic polymeric nanoparticles. Eur J Pharm Sci.

[5] Dilnawaz F, Singh A, Mohanty C, Sahoo SK (2010). Dual drug loaded superparamagnetic iron oxide nanoparticles for targeted cancer therapy. Biomaterials.

[6] Kumar CS, Mohammad F (2011). Magnetic nanomaterials for hyperthermia-based therapy and controlled drug delivery. Adv Drug Deliv Rev.

[7] Ji X, Shao R, Elliott AM, Stafford RJ, Esparza-Coss E, Bankson JA (2007). Bifunctional gold nanoshells with a superparamagnetic iron oxide-silica core suitable for both mr imaging and photothermal therapy. J Phys Chem C Nanomater Interfaces.

[8] Thomas R, Park IK, Jeong YY (2013). Magnetic iron oxide nanoparticles for multimodal imaging and therapy of cancer. Int J Mol Sci.

[9] Hsieh WJ, Liang CJ, Chieh JJ, Wang SH, Lai IR, Chen JH (2012). In vivo tumor targeting and imaging with anti-vascular endothelial growth factor antibody-conjugated dextran-coated iron oxide nanoparticles. Int J Nanomedicine.

[10] Chan LW, Wang YN, Lin LY, Upton MP, Hwang JH, Pun SH (2013). Synthesis and characterization of anti-egfr fluorescent nanoparticles for optical molecular imaging. Bioconjug Chem.

[11] Jiang J, Dong L, Wang L, Wang L, Zhang J, Chen F (2016). Her2-targeted antibody drug conjugates for ovarian cancer therapy. Eur J Pharm Sci.

[12] Sugahara KN, Teesalu T, Karmali PP, Kotamraju VR, Agemy L, Greenwald DR (2010). Coadministration of a tumor-penetrating peptide enhances the efficacy of cancer drugs. Science.

[13] Hernandez FJ, Hernandez LI, Pinto A, Schäfer T, Özalp VC (2013). Targeting cancer cells with controlled release nanocapsules based on a single aptamer. Chem Commun (Camb).

[14] Pavlov V, Xiao Y, Shlyahovsky B, Willner I (2004). Aptamer-functionalized au nanoparticles for the amplified optical detection of thrombin. J Am Chem Soc.

[15] Tong R, Yala L, Fan TM, Cheng J (2010). The formulation of aptamer-coated paclitaxel-polylactide nanoconjugates and their targeting to cancer cells. Biomaterials.

[16] Li X, Zhao Q, Qiu L (2013). Smart ligand: Aptamer-mediated targeted delivery of chemotherapeutic drugs and sirna for cancer therapy. J Control Release.

[17] Kim D, Jeong YY, Jon S (2010). A drug-loaded aptamer-gold nanoparticle bioconjugate for combined CT imaging and therapy of prostate cancer. ACS Nano.

[18] Jalalian SH, Taghdisi SM, Shahidi Hamedani N, Kalat SA, Lavaee P, ZandKarimi M (2013). Epirubicin loaded super paramagnetic iron oxide nanoparticle-aptamer bioconjugate for combined colon cancer therapy and imaging in vivo. Eur J Pharm Sci.

[19] Liu Y, Li K, Pan J, Liu B, Feng SS (2010). Folic acid conjugated nanoparticles of mixed lipid monolayer shell and biodegradable polymer core for targeted delivery of docetaxel. Biomaterials.

[20] Sun C, Sze R, Zhang M (2006). Folic acid-PEG conjugated superparamagnetic nanoparticles for targeted cellular uptake and detection by MRI. J Biomed Mater Res A.

[21] Kim JK, Choi KJ, Lee M, Jo MH, Kim S (2012). Molecular imaging of a cancer-targeting theragnostics probe using a nucleolin aptamer- and microrna-221 molecular beacon-conjugated nanoparticle. Biomaterials.

[22] Ferreira CS, Cheung MC, Missailidis S, Bisland S, Gariépy J (2009). Phototoxic aptamers selectively enter and kill epithelial cancer cells. Nucleic Acids Res.

[23] Azhdarzadeh M, Atyabi F, Saei AA, Varnamkhasti BS, Omidi Y, Fateh M (2016). Theranostic muc-1 aptamer targeted gold coated superparamagnetic iron oxide nanoparticles for magnetic resonance imaging and photothermal therapy of colon cancer. Colloids Surf B Biointerfaces.

[24] Xu Z, Hou Y, Sun S (2007). Magnetic core/shell fe3o4/au and fe3o4/au/ag nanoparticles with tunable plasmonic properties. J Am Chem Soc.

[25] Mahmoudi M, Abdelmonem AM, Behzadi S, Clement JH, Dutz S, Ejtehadi MR (2013). Temperature: The “ignored” factor at the nanobio interface. ACS Nano.

[26] Veiseh O, Gunn JW, Zhang M (2010). Design and fabrication of magnetic nanoparticles for targeted drug delivery and imaging. Adv Drug Deliv Rev.

[27] Kayal S, Ramanujan RV (2010). Doxorubicin loaded PVA coated iron oxide nanoparticles for targeted drug delivery. Mater Sci Eng C.

[28] Ye F, Barrefelt Å, Asem H, Abedi-Valugerdi M, El-Serafi I, Saghafian M (2014). Biodegradable polymeric vesicles containing magnetic nanoparticles, quantum dots and anticancer drugs for drug delivery and imaging. Biomaterials.

[29] Lee SJ, Lee HJ, Moon MJ, Vu-Quang H, Lee HJ, Muthiah M (2011). Superparamagnetic iron oxide nanoparticles-loaded polymersome-mediated gene delivery guided by enhanced magnetic resonance signal. J Nanosci Nanotechnol.

[30] Qin J, Laurent S, Jo YS, Roch A, Mikhaylova M, Bhujwalla ZM (2007). A high-performance magnetic resonance imaging T2 contrast agent. Adv Mater.

[31] Ma X, Gong A, Chen B, Zheng J, Chen T, Shen Z (2015). Exploring a new SPION-based MRI contrast agent with excellent water-dispersibility, high specificity to cancer cells and strong MR imaging efficacy. Colloids Surf B Biointerfaces.

[32] Bull E, Madani SY, Sheth R, Seifalian A, Green M, Seifalian AM (2014). Stem cell tracking using iron oxide nanoparticles. Int J Nanomedicine.

[33] Laurent S, Dutz S, Häfeli UO, Mahmoudi M (2011). Magnetic fluid hyperthermia: Focus on superparamagnetic iron oxide nanoparticles. Adv Colloid Interface Sci.

[34] Hayashi K, Nakamura M, Sakamoto W, Yogo T, Miki H, Ozaki S (2013). Superparamagnetic nanoparticle clusters for cancer theranostics combining magnetic resonance imaging and hyperthermia treatment. Theranostics.

[35] Silva AC, Oliveira TR, Mamani JB, Malheiros SM, Malavolta L, Pavon LF (2011). Application of hyperthermia induced by superparamagnetic iron oxide nanoparticles in glioma treatment. Int J Nanomedicine.

[36] Madani F, Esnaashari SS, Mujokoro B, Dorkoosh F, Khosravani M, Adabi M (2018). Investigation of effective parameters on size of paclitaxel loaded plga nanoparticles. Adv Pharm Bull.

[37] Kouassi GK, Irudayaraj J (2006). Magnetic and gold-coated magnetic nanoparticles as a DNA sensor. Anal Chem.

[38] Lyon JL, Fleming DA, Stone MB, Schiffer P, Williams ME (2004). Synthesis of fe oxide core/au shell nanoparticles by iterative hydroxylamine seeding. Nano Lett.

[39] Sun C, Lee JS, Zhang M (2008). Magnetic nanoparticles in MR imaging and drug delivery. Adv Drug Deliv Rev.

[40] Bogart LK, Taylor A, Cesbron Y, Murray P, Lévy R (2012). Photothermal microscopy of the core of dextran-coated iron oxide nanoparticles during cell uptake. ACS Nano.

[41] Blackburn MJ, Wheldon TE, Field SB, Goldman JM (1984). The sensitivity to hyperthermia of human granulocyte/macrophage progenitor cells (CFU-GM) derived from blood or marrow of normal subjects and patients with chronic granulocytic leukaemia. Br J Cancer.

[42] Lu AH, Salabas EL, Schüth F (2007). Magnetic nanoparticles: Synthesis, protection, functionalization, and application. Angew Chem Int Ed Engl.

[43] Kang B, Mackey MA, El-Sayed MA (2010). Nuclear targeting of gold nanoparticles in cancer cells induces DNA damage, causing cytokinesis arrest and apoptosis. J Am Chem Soc.

[44] Markovic ZM, Harhaji-Trajkovic LM, Todorovic-Markovic BM, Kepić DP, Arsikin KM, Jovanović SP (2011). In vitro comparison of the photothermal anticancer activity of graphene nanoparticles and carbon nanotubes. Biomaterials.

[45] Tong L, Zhao Y, Huff TB, Hansen MN, Wei A, Cheng JX (2007). Gold nanorods mediate tumor cell death by compromising membrane integrity. Adv Mater.

